# Expanded description and distribution of *Opisthotropis tamdaoensis* Ziegler, David & Vu, 2008 (Squamata, Natricidae), first record from Yunnan Province of China

**DOI:** 10.3897/BDJ.14.e181301

**Published:** 2026-01-15

**Authors:** Siyuan Zhao, Dingding Ou, Long Zhang, Bo Cai, Shun Ma, Shuo Liu, Keji Guo, Wei Zhou

**Affiliations:** 1 Central South Forest Inventory and Planning Institute of State Forestry Administration, Changsha, China Central South Forest Inventory and Planning Institute of State Forestry Administration Changsha China; 2 China-Croatia Belt and Road Joint Laboratory on Biodiversity and Ecosystem Services, Chengdu Institute of Biology, Chinese Academy of Sciences, Chengdu, China China-Croatia Belt and Road Joint Laboratory on Biodiversity and Ecosystem Services, Chengdu Institute of Biology, Chinese Academy of Sciences Chengdu China; 3 University of Chinese Academy of Science, Beijing, China University of Chinese Academy of Science Beijing China; 4 Kunming Natural History Museum of Zoology, Kunming Institute of Zoology, Chinese Academy of Sciences, Kunming, China Kunming Natural History Museum of Zoology, Kunming Institute of Zoology, Chinese Academy of Sciences Kunming China; 5 Yunnan Key Laboratory of Biodiversity Information, Kunming Institute of Zoology, Chinese Academy of Sciences, Kunming, China Yunnan Key Laboratory of Biodiversity Information, Kunming Institute of Zoology, Chinese Academy of Sciences Kunming China

**Keywords:** new national record, Tam Dao Mountain Stram Keelback, molecular analysis, morphological characters, Yunnan, China

## Abstract

**Background:**

The Tam Dao Mountain Stream Keelback, *Opisthotropis tamdaoensis* Ziegler, David & Vu, 2008, was previously described, based on one single holotype and subsequently expanded according to four newly-collected specimens from the type locality. Information on this species is extremely limited.

**New information:**

On the basis of newly-collected *Opisthotropis* specimens from Hekou, Yunnan, China, this study reports the first country record of *Opisthotropis tamdaoensis* from China. The Chinese *Opisthotropis tamdaoensis* population totally agrees with the original description of this species and revealed only a minor genetic distance (lower than 2.9%) in the mitochondrial gene fragment of the cytochrome *b* (*Cytb*) compared with the topotypic population. Based upon the our newly-collected specimens and published data, we revised the diagnosis of *Opisthotropis tamdaoensis*:

1. nasal not divided below nostril;
2. loreal usually 1, not in contact with internasals;
3. pre-oculars 1–2, postoculars 2 subocular, usually 1;
4. anterior temporals 1–2, posterior temporals 2–4;
5. supralabials usually 9, only the fifth or fifth to sixth supralabial (rarely none) in contact with the orbit;
6. infralabials usually 9–10;
7. first pair of chin shields longer than the second pair;
8. dorsal scales smooth anteriorly, keeled posteriorly, in (17–19)-17-17 rows;
9. tails relatively short, TaL/TL 0.143–0.161;
10. pre ventrals 0–4, ventrals 160–176, precloacal divided; 48–56 divided subcaudals;
11. uniform olive-grey dorsum, with a dark longitudinal lateral stripe within the dark flank colouration, ventral side pale or light yellow, without sharp transition towards the dark dorsal colouration, subcaudal region may bear dark mottling.

This finding broadly extends our understanding of this poorly-known snake species and raises the total known *Opisthotropis* number in China to 14. Moreover, this study reports the second *Opisthotropis* species in Yunnan Province, China.

## Introduction

Mountain Stream Keelbacks, *Opisthotropis* Günther, 1872, are a group of small, nocturnal, aquatic snakes, currently including 25 species, mainly distributed across southern China and Southeast Asia and extending to the Ryukyu Archipelago of Japan, Sumatra of Indonesia and the Philippines (Gao et al. 2024, Ren et al. 2025, Uetz et al. 2025). Recent studies revealed a rich taxonomic diversity in *Opisthotropis*, but many species remained enigmatic and poorly studied (Ren et al. 2017, Wang et al. 2017, Ziegler et al. 2018, Ziegler et al. 2019, Wang et al. 2020). Amongst the recognised *Opisthotropis* species, we have recorded 13 in China presently, respectively: *O. andersonii* (Boulenger, 1888), *O. cheni* Zhao, 1999, *O. guangxiensis* Zhao, Jiang & Huang, 1978, *O. haihaensis* Ziegler, Pham, Nguyen, Nguyen, Wang, Wang, Stuart & Le, 2019, *O. hungtai* Wang, Lyu, Zeng, Lin, Yang, Nguyen, Le, Ziegler & Wang, 2020, *O. jacobi* Angel & Bourret, 1933, *O. kuatunensis* Pope, 1928, *O. lateralis* Boulenger, 1903, *O. latouchii* (Bouleger, 1899), *O. laui* Yang, Sung & Chan, 2013, *O. maxwelli* Boulenger, 1914, *O. shenzhenensis* Wang, Guo, Liu, Lyu, Wang, Luo, Sun & Zhang, 2017 and *O. zhaoermii* Ren, Wang, Jiang, Guo & Li, 2017 (Wang et al. 2020, Jiang et al. 2025, Zhang et al. 2025).

In the past, *Opisthotropis tamdaoensis* Ziegler, David & Vu, 2008 was only described, based on a single specimen (VNUH 010606) and morphological data (Ziegler et al. 2008) and then some further specimens were supplied; moreover, its phylogenetic relationships were also detected, supporting the species validity (Ziegler et al. 2015, Ziegler et al. 2017). However, this species remained inadequately studied and only found in a small zone near its type locality, Tam Dao in Vietnam.

During our herpetological survey in Hekou Yao Autonomous County, Yunnan Province, China, two specimens of *Opisthotropis* and one sample tissue from a released individual were collected. Subsequent morphological and molecular analyses indicate that these specimens belong to *O. tamdaoensis*. Thus, we here report the first national record of *O. tamdaoensis* in China. Additionally, there is only one *Opisthotropis* species, *O. jacobi*, recorded in Yunnan Province (Wang et al. 2022) and the other one previously documented species *O. praemaxillaris* (Angel, 1929) (Cai et al. 2015) has been transferred to *Paratapinophis praemaxillaris*, which is now revised as *Trimerodytes praemaxillaris* (Murphy et al. 2008, David et al. 2015, Ren et al. 2018, Ren et al. 2019, Deepak et al. 2021), so this discovery also represents the second provincial *Opisthotropis* species record from Yunnan Province, China.

## Materials and methods

**Materials**

**Figure 1.**
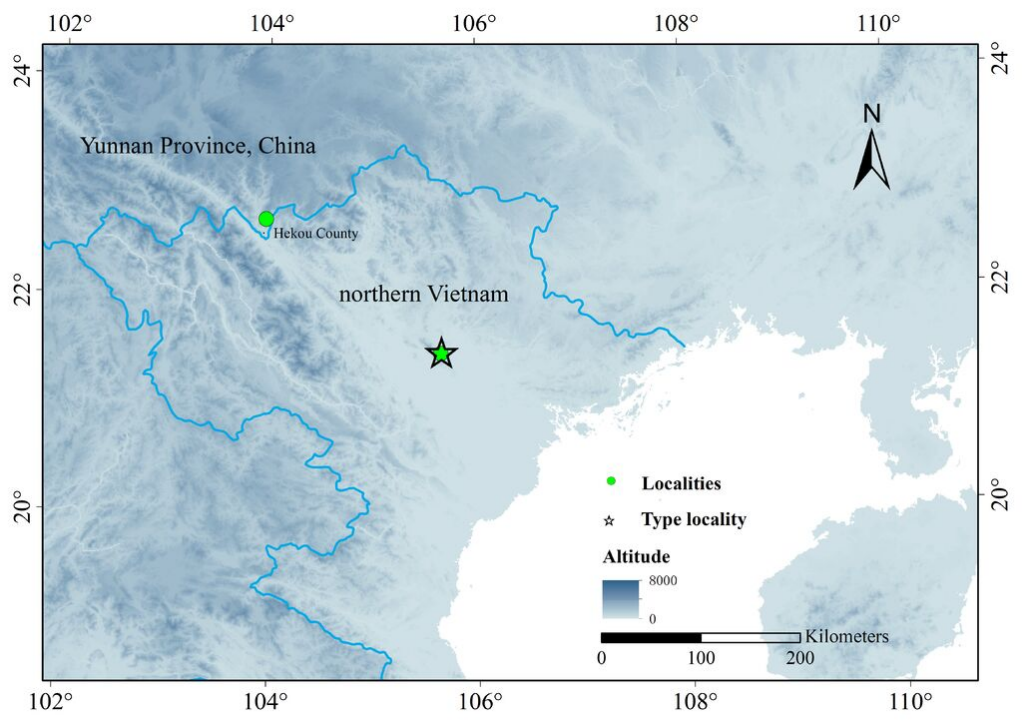
Distribution sites of *Opisthotropis tamdaoensis*, the star presenting the type locality, Tam Dao in Vietnam and the green dot presenting the new country record.

Two *Opisthotropis* specimens (CIB 103778 and CIB 103779) and one tissue sample (sampling from a released snake) (YN-HKX-202510-1) from Hekou Yao Autonomous County, Honghe Hani and Yi Autonomous Prefecture, Yunnan Province, China (22.688042°N, 103.960274°E, 152m) were collected (Fig. 1). The collected specimens were preserved in 75% ethanol and after, fixed in 95% ethanol for one day and their tissue samples were extracted and preserved in 95% ethanol. These specimens were deposited in the Chengdu Institute of Biology (CIB), Chinese Academy of Sciences (CAS) and the Central South Forest Inventory and Planning Institute of State Forestry Administration (CSAIPN), respectively.

**Molecular data and phylogenetic analysis**

Total genomic DNA was extracted by Vazyme FastPure Blood/Cell/Tissue/Bacteria DNA Isolation Mini Kit (Vazyme Biotech Co., Ltd, Nanjing, China) from the tissue samples of each specimen. One mitochondrial gene fragment of cytochrome *b* (*Cytb*) was targeted and amplified by the polymerase chain reaction (PCR) using the primers L14910 (5’-GACCTGTGATMTGAAAACCAYCGTTGT-3’) and H16064 (5’-CTTTGGTTTACAAGAACAATGCTTTA-3’) (Burbrink et al. 2000). PCR was performed in 25 μl reactant with the following cycling conditions: first an initial denaturing step at 94°C for 7 min; then 41 cycles of denaturing at 94°C for 40 s, annealing at 46°C for 30 s and extending at 72°C for 60 s; lastly, a final extending step at 72°C for 8 min (Gao et al. 2024). PCR products were sequenced by Beijing Qingke New Industry Biotechnology Co., Ltd

A total of 40 sequences were used for phylogenetic analysis, amongst which 38 sequences were from 18 *Opisthotropis* species and the remining two species were from the outgroups, *Trimerodytes balteatus* and *T. percarinatus* (Ren et al. 2019) and all sequences’ information can be found in Table 1. All sequences (1,051 bp) were input in MEGA11 (Tamura et al. 2021) and aligned by MUSCLE (Edgar 2004) and the uncorrected pairwise distances (*p*-distance) were calculated in MEGA11. The Maximum Likelihood (ML) analysis was performed in IQ-TREE 1.6.12 (Nguyen et al. 2014) based on the best-fit model TPM2+F+I+G4 computed by ModelFinder for IQ-Tree in PhyloSuite 1.2.3 according to Bayesian Information Criterion (BIC) (Kalyaanamoorthy et al. 2017, Zhang et al. 2019). Ultrafast Bootstrap Approximation (UFB) nodal support was assessed by using ten thousand ultrafast bootstrap replicates and when the value (UFB, %) was ≥ 95, it would be considered as significantly supported (Hoang et al. 2017). The single branch tests were conducted by SH-like approximate likelihood ratio test (SH-aLRT) by 1000 replicates and when the nodal support (SH, %) is ≥ 80, it would also be considered well supported (Stéphane et al. 2010). The Bayesian Inference (BI) analysis was conducted via MrBayes 3.2.1 (Ronquist et al. 2012) under the best-fit model GTR+F+I+G4, which was calculated according to BIC as well by ModelFinder for MrBayes in PhyloSuite 1.2.3. The BI analysis programme worked through two independent runs with a four-chain run calculated for 10 million generations using the Markov Chain Monte Carlo (MCMC), sampling every 1000 with the first 25% of samples discarded as burn-in and resulting in a potential scale reduction factor (PSRF) of < 0.005. The nodal support Bayesian posterior probabilities (BI, %) ≥ 95 were considered significantly supported.

**Morphology**

Morphological measurements and scalation features followed Zhao (2006), Wang et al. (2017) and Ziegler et al. (2019). Measurements contained: TL (total length), SVL (snout-vent length), TaL (tail length), HL (head length) and HW (head width), amongst which TL, SVL and TaL were measured to the nearest 1 mm by Deli Stainless Ruler (No. 8460) and HL and HW were measured to the nearest 0.01 mm by Deli Digital Vernier Caliper (DL91150). Scalation features containing PrO (pre-oculars), PtO (postoculars), SPL (supralabials), SPL-Orbit (the numbers of SPL touching the orbit), IFL (infralabials), TMP (temporals), V (ventral scales), SC (subcaudals) and DSR (dorsal scale rows) were counted at one head length behind head, at mid-body and at one head length before vent, respectively. Bilateral scale counts were given as left/right. The morphological data of published *O. tamdaoensis* specimens were obtained from literature (Ziegler et al. 2008, Ziegler et al. 2017).

## Taxon treatments

### Opisthotropis tamdaoensis

Ziegler, David & Vu, 2008

C8E18205-05DD-5683-A5F8-06272357A339

31C74129-7B4E-49F5-B141-6A46D698CF76

#### Materials

- **Type status:** Other material. **Occurrence:** lifeStage: juvenile; occurrenceID: D9E2B425-59B2-5B33-89A7-44A75C710694; **Taxon:** scientificName: *Opisthotropis tamdaoensis*; class: Reptilia; order: Squamata; family: Natricidae; genus: Opisthotropis; specificEpithet: *tamdaoensis*; scientificNameAuthorship: Ziegler, David & Vu, 2008; **Location:** country: China; stateProvince: Yunnann; county: Hekou Yao Autonomous County; locality: Baisha River, Nanxi Town; verbatimElevation: 152 m; decimalLatitude: 22.688042; decimalLongitude: 103.960274; **Event:** eventDate: 11 April 2015; fieldNumber: CIB 103778
- **Type status:** Other material. **Occurrence:** sex: female; lifeStage: adult; occurrenceID: F5AC49FC-216E-571D-9AC2-384C90F2F50D; **Taxon:** scientificName: *Opisthotropis tamdaoensis*; class: Reptilia; order: Squamata; family: Natricidae; genus: Opisthotropis; specificEpithet: *tamdaoensis*; scientificNameAuthorship: Ziegler, David & Vu, 2008; **Location:** country: China; stateProvince: Yunnan; county: Hekou Yao Autonomous County; locality: Baisha River, Nanxi Town; verbatimElevation: 152 m; decimalLatitude: 22.688042; decimalLongitude: 103.960274; **Event:** eventDate: 11 April, 2015; fieldNumber: CIB 103779

#### Etymology

According to the original description, the specific name “*tamdaoensis*” refers to the type locality of this species, Tam Dao Mountain ridge (Vinh Phuc Province) in Vietnam (Ziegler et al. 2008). The English name was “Tam Dao Mountain Stream Snake”. As this species is now also reported from China, we suggest its Chinese name as “三岛后棱蛇” (Sān Dăo Hòu Léng Shé), derived from its scientific name.

#### Description of the specimens from China (n = 2)

The morphological measurements and scalation features are listed in Table 2. Body medium size, TL 558 mm (except the juvenile), cylindrical, slenderly built; head short and broad, HL/HW 1.18, dorsally depressed, indistinct from neck (except the juvenile); snout moderate; pupil round; tail relatively short, TaL/TL 0.161–0.182, tapering posteriorly.

**Head scalation.** Rostral arc, broad, approximately twice as long as high, visible from above. Internasal two, crescent-shaped, in contact with each other medially behind the rostral, not in contact with loreal, posteriorly in contact with prefrontal. Prefrontal one, in contact with internasal, nasal, loreal, pre-ocular, frontal and supraocular. Frontal one, sub-hexagonal, in contact with supra-ocular and parietals. Parietals large and in contact medially. Nasal directed dorsally, polygonal, not divided, in contact with rostral anteriorly, with the 1^st^ and 2^nd^ supralabials ventrally, with loreal and prefrontal posteriorly and with internasal dorsally. Nostril long, oval, piecing in middle of nasal, oblique, directed upwards. Loreal one, not entering the orbit, in contact with the 2^nd^, 3^rd^ and 4^th^ supralabials. Pre-ocular one, height larger than width, in contact with the 5^th^ supralabial. Supra-ocular one, nearly twice as long as wide. Postoculars two, the upper one much larger than the lower one. A single anterior temporal (rarely two) and two posterior temporals. Supralabials nine (rarely eight) and the fourth and fifth supralabials entering the orbit. Infralabials nine (rarely ten), the first one in contact with each other behind the mental. Two pairs of chin shields, anterior chin shields larger than the posterior chin shields.

**Body scalation.** Dorsal scale rows 17:17:17, dorsal scales of the anterior body smooth and of the posterior body keeled. Ventrals 160–165. Cloacal plate divided. Subcaudals 51–56, paired.

**Figure 2.**
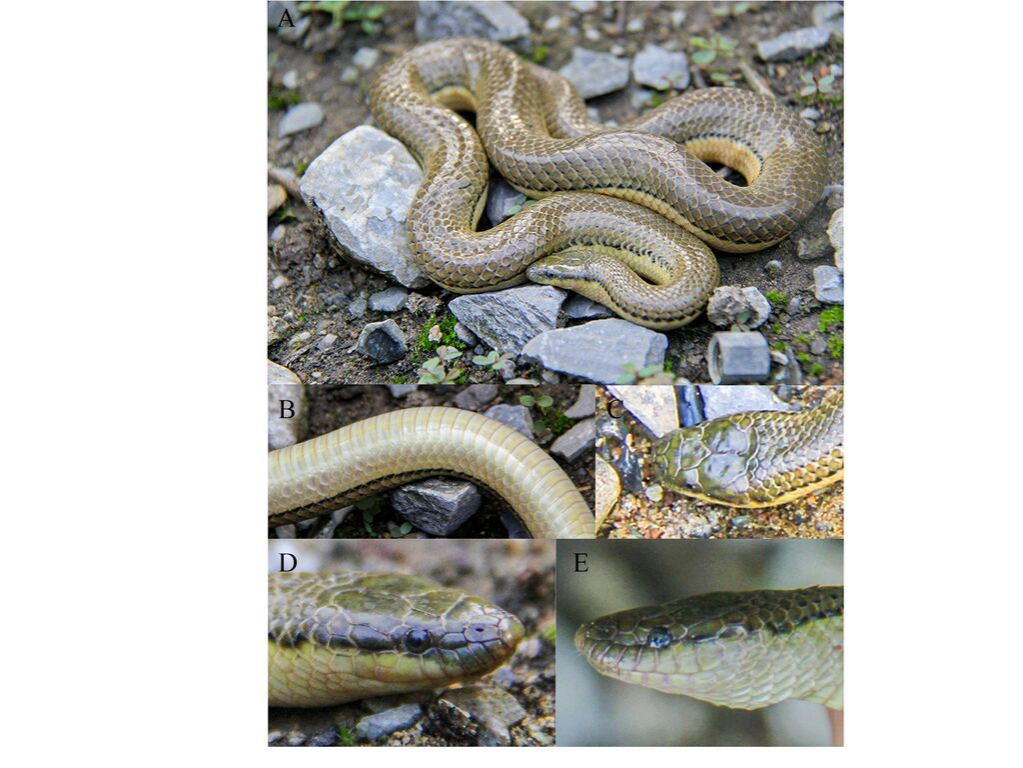
Photographs of the adult female *Opisthotropis tamdaoensis* specimen from Yunnan, China (CIB 103779) in life. **A** Total view; **B** Ventral view; **C** Dorsal view of head; **D** right view of head; **E** Left view of head. Photos by Bo Cai.

**Colouration in life.** (Fig. 2) Eye brown, pupil black; scales on dorsal surface of head olive grey with scattered dark green patches; chin shields light yellow with brownish-black mottling at each margin; body olive-grey with black spots on each junction of the dorsal scales, black spots becoming larger on sides of body and connecting into a side line pattern and the dorsal scales below the lateral lines turning to light yellow; ventrals light yellow; tail olive-grey also with black spots on each junction of the dorsal scales, black spots becoming larger on sides of tail and connecting into a side line pattern and faded at the tail tip; subcaudals light yellow with discontinuous black scattered mottling.

**Figure 3.**
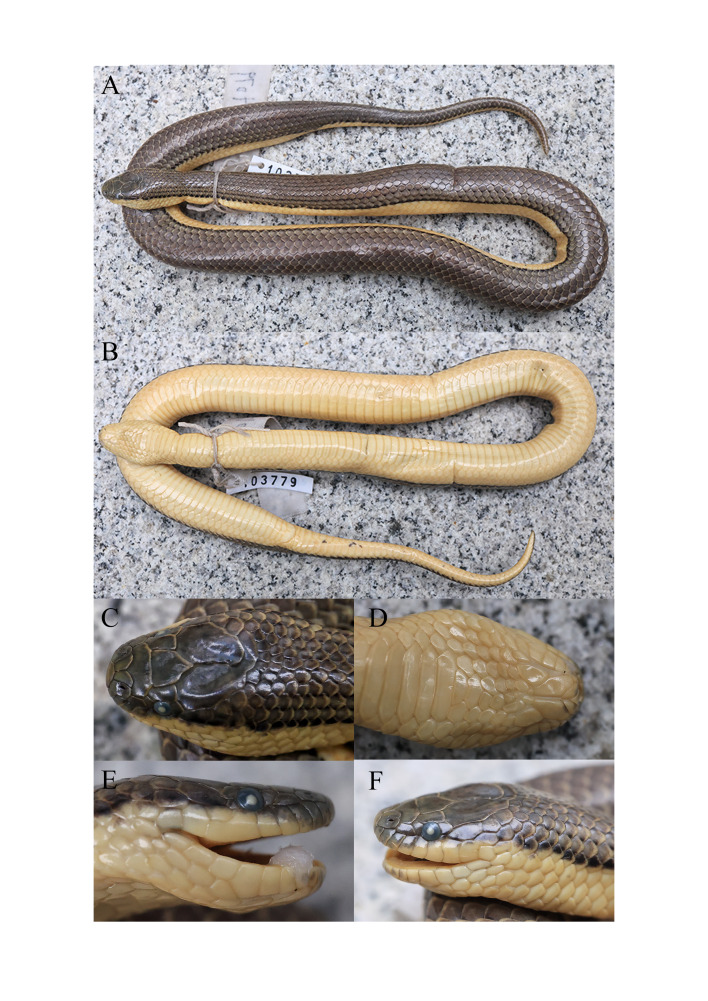
Photographs of the adult female *Opisthotropis tamdaoensis* specimen from Yunnan, China (CIB 103779) in preservative. **A** Total view; **B** Ventral view; **C** Dorsal view of head; **D** Ventral view of head; **E** right view of head; **F** Left view of head. Photos by Bo Cai.

**Figure 4.**
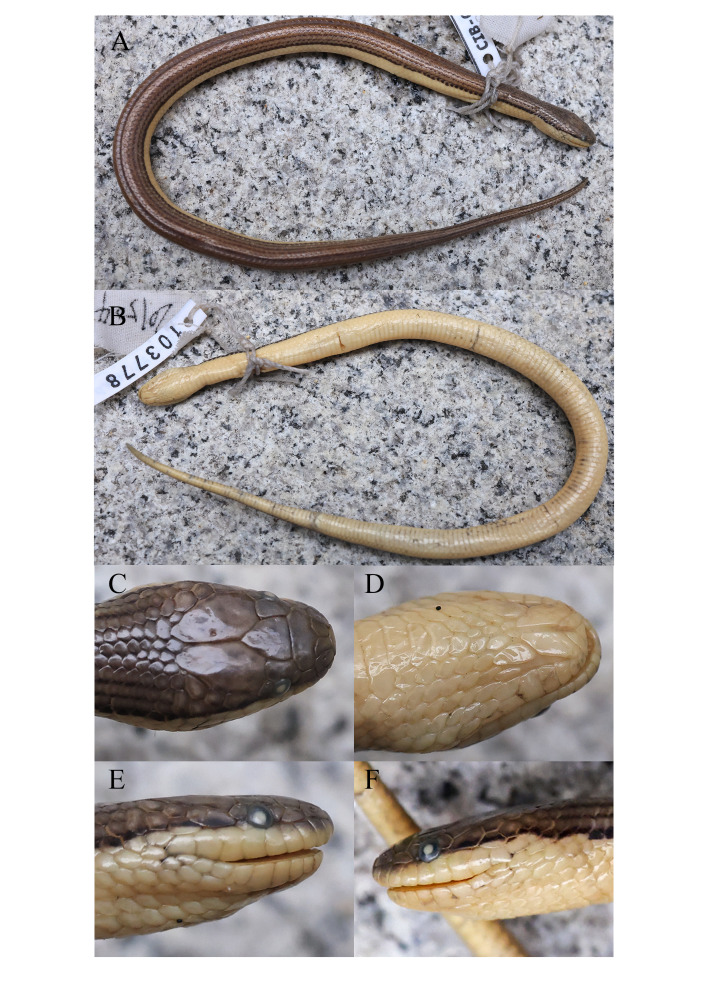
Photographs of the juvenile *Opisthotropis tamdaoensis* specimen from Yunnan, China (CIB 103778) in preservative. **A** Total view; **B** Ventral view; **C** Dorsal view of head; **D** right view of head; **E** Left view of head. Photos by Bo Cai.

**Colouration in preservation.** (Figs 3, 4) Scales on dorsal surface of head brown with scattered dark patches; chin shields light creamy-yellow with brownish-black mottling at each margin; body olive-brown with black spots on each junction of the dorsal scales, black spots becoming larger on sides of body and connecting into a side line pattern and the dorsal scales below the lateral lines turning to light creamy-yellow; ventrals light creamy-yellow; tail olive-brown also with black spots on each junction of the dorsal scales, black spots becoming larger on sides of tail and connecting into a side line pattern and fading at the tail tip; subcaudals light creamy-yellow with discontinuous black scattered mottling.

#### Extended diagnosis

(1) Nasal not divided below nostril; (2) loreal usually 1, not in contact with internasals; (3) pre-oculars 1–2, postoculars 2 subocular, usually 1; (4) anterior temporals 1–2, posterior temporals 2–4; (5) supralabials usually 9, only the fifth or fifth to sixth supralabial (rarely none) in contact with the orbit; (6) infralabials usually 9–10; (7) first pair of chin shields longer than the second pair; (8) dorsal scales smooth anteriorly, keeled posteriorly, in (17–19)-17-17 rows; (9) tails telatively short, TaL/TL 0.143–0.161; (10) pre ventrals 0–4, ventrals 160–176, precloacal divided; 4856 divided subcaudals; (11) uniform olive-grey dorsum, with a dark longitudinal lateral stripe within the dark flank colouration, ventral side pale or light yellow, without sharp transition towards the dark dorsal colouration, subcaudal region may bear dark mottling.

#### Distribution

*O. tamdaoensis* is currently only known from two localities, where one is from its type localities, Silver Stream in Tam Dao, Vinh Phuc Province, northern Vietnam and the other one is from Baisha River, Nanxi Town, Hekou Yao Autonomous County, Honghe Hani and Yi Autonomous Prefecture, Yunnan Province, China. The Vietnamese specimens were found at elevations in between 750 to 1,500 m above sea level and the Chinese specimens were found at 152 m above sea level, thus the elevation range of *O. tamdaoensis* is from 152 m to 1,500 m a.s.l. (Ziegler et al. 2008, Ziegler et al. 2017).

#### Natural history notes

*O. tamdaoensis* inhabits in secondary monsoon tropical evergreen forest on granitic soil or karst landscapes. The Vietnamese specimens were found between 09:00 to 12:00 am and the Chinese specimens were found at night in April and October, respectively. The snakes were in the shallow water in stream sections with open forest and we also found *Odorrana cf. graminea* (Boulenger, 1899), *Cyrtodactylus gulinqingensis* Liu, Li, Hou, Orlov & Ananjeva, 2021 and *Calotes emma* Gray, 1845 as co-occurring with the Chinese records (Ziegler et al. 2017).

## Analysis

**Figure 5.**
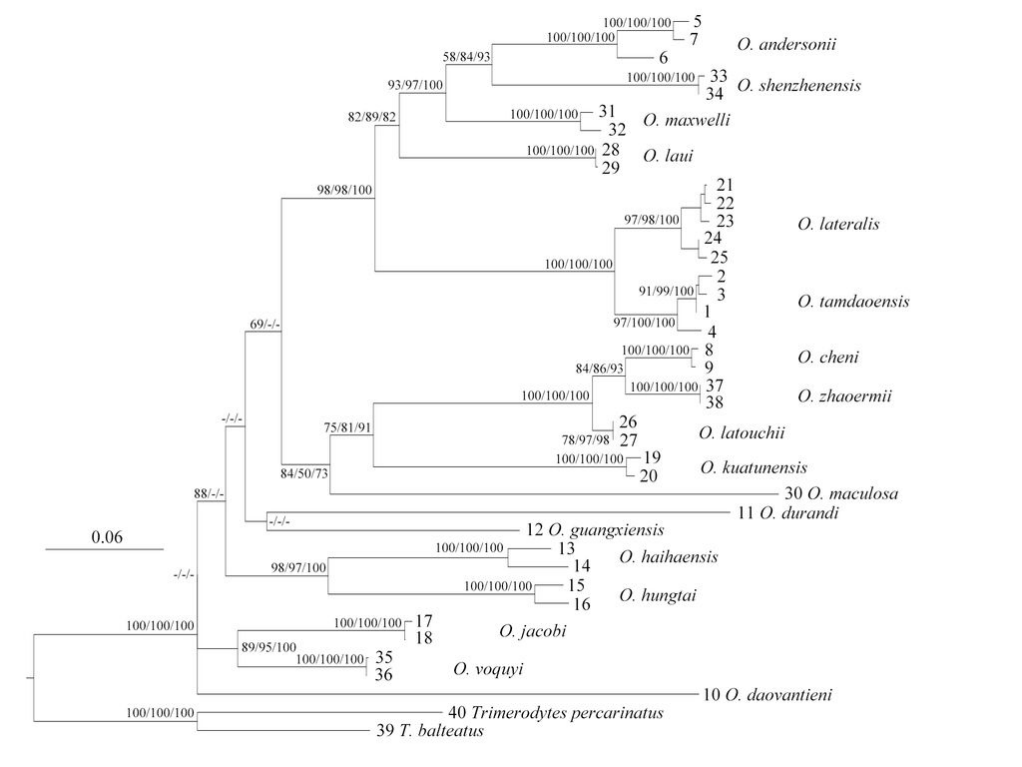
Maximum Likelihood tree topology of *Opisthotropis* inferred from *Cytb* (1,051 bp). The support values of each node present on the tree: SH / UFB / BI (the nodes lower than 50 are displayed as “-”). Numbers refer to specimens listed in Table 1.

Based on our phylogenetic analysis using *Cytb* (1,051 bp) (Fig. 5), all *Opisthotropis* specimens clustered into a monophyletic group with high nodal supports (SH 100 / UFB 100 / BI 100), although the relationships amongst some species remained unresolved. The newly-collected Chinese *Opisthotropis* specimens nested with *O. tamdaoensis* (SH 100 / UFB 100 / BI 100) with a small genetic divergence (0.5%–2.9%), then forming a sister clade to *O. lateralis* with a distinct genetic distance (5.9%–6.7%) (Table 3). The genetic differentiation within *O. tamdaoensis* is clearly lower than the intraspecific differentiation of other known *Opisthotropis* species (4.5% in *O. andersonii*, 4.4% in *O. haihaensis*), in support of its intraspecific variation. This supports the identification of the newly-discovered Chinese *Opisthotropis* population as *O. tamdaoensis*.

Compared to the morphological descriptions available for *O. tamdaoensis* specimens from Vietnam (Ziegler et al. 2008, Ziegler et al. 2017), the Chinese specimens possess a longer tail (TaL/TL 0.161–0.182 vs. 0.143–0.157), fewer ventrals (CIB 103779 160 vs. 162–176), more subcaudals (SC 51–56 vs. 48–51) and fewer anterior dorsal scale rows (17 vs. 19). We interpret these few slightly different characters as intraspecific variation and, thus, extension of the original diagnosis, as so far, only few indviduals of this snake species have been known to science (Table 2). Therefore, when combining the molecular and morphological evidences, the newly-collected *Opisthotropis* specimens from China can be identified as *O. tamdaoensis* and, thus, herein we revise and extend the diagnosis of *O. tamdaoensis* according to the newly-collected and published specimens and report it as a new record for China.

## Discussion

China possesses a high *Opisthotropis* diversity, mainly distributed in southern, eastern and central China and the species diversity may still be underestimated (Ren et al. 2025). The discovery of *O. tamdaoensis* raises the total *Opisthotropis* number of China to 14, broadly expanding our understanding of this genus. Moreover, according to the updated reptile checklist of Yunnan Province (Wang et al. 2022), only one *Opisthotropis* species was documented and our research revealed *O. tamdaoensis* was the second *Opisthotropis* species in Yunnan. The hemipenial morphology plays a key diagnostic character for natricine snakes (Gao et al. 2024). However, we only collected one female and one juvenile specimen, thus we could not examine the hemipenial morphology of this species, which should become a target in the future study. The phylogenetic relationships of *Opisthotropis* remain unresolved (Fig. 5), based upon only a single mitochondrial gene fragment (Cytb), although our results can support this identification, but a future phylogenetic analysis using genomic datasets is needed to clarify the *Opisthotropis* relationships. More investigation and phylogenetic research of this genus still need to be conducted.

*O. tamdaoensis* was previously considered as an enigmatic species and only found around its type locality, Tam Dao in northern Vietnam. Our finding explored a new locality of this species, where the straight-line distance is approximately 210 km. Therefore, *O. tamdaoensis* is currently known to inhabit the area from Tam Dao (Vinh Phuc Province, Vietnam) to our new site, Hekou (Yunnan Province, China), with an elevation range from 150 m to 1,500 m above sea level (Ziegler et al. 2008, Ziegler et al. 2017). Future investigation should be conducted to recognise the species population’s connectivity and status.

Hekou Yao Autonomous County, located in the southern Yunnan, possesses a high herpetological diversity pattern, which was probably still underestimated. In recent years, dozens of new species or new national records were found in Hekou and its nearby regions, i.e. (1) amphibians: *Amolops compotrix* (Bain, Stuart, & Orlov, 2006) (Huang et al. 2025), *Amolops shihaitaoi* Wang, Li, Du, Hou & Yu, 2022 (Wang et al. 2022), *Leptobrachella pingbianensis* (Rao, Hui, Zhu & Ma, 2022) (Rao 2022) and some others (Liu et al. 2021a, Du et al. 2022, Liu et al. 2022, Wang et al. 2023, Du et al. 2024, Liu et al. 2025, Pan et al. 2025); (2) reptiles: *Achalinus pingbianensis* Li, Yu, Wu, Liao, Tang, Liu & Guo, 2020 (Li et al. 2020), *Cyrtodactylus gulinqingensis* Liu, Li, Hou, Orlov & Ananjeva, 2021 (Liu et al. 2021b), *Ovophis anitae* David, Frétey & Vogel, 2024 (Zeng et al. 2023, David et al. 2024), *Tropidophorus vongx* Wang, Li, Mu, Xu & Che, 2024 (Wang et al. 2024). These new findings of amphibians and reptiles point to a biodiversity hotspot, which requires further conservation measures.

## Supplementary Material

XML Treatment for Opisthotropis tamdaoensis

## Figures and Tables

**Table 1.** Samples of *Opisthotropis* and the outgroup *Trimerodytes* used in this study.

No.	Species	Voucher No.	Locality	GenBank No.	References
1	*O. tamdaoensis*	YN-HKX-202510-1	China, Yunnan, Hekou	PX829133	This study
2	*O. tamdaoensis*	CIB 103778	China, Yunnan, Hekou	PX829134	This study
3	*O. tamdaoensis*	CIB 103779	China, Yunnan, Hekou	PX829135	This study
4	*O. tamdaoensis*	IEBR A.2016.33	Vietnam, Vinh Phuc, Tam Dao	MF477901	Ziegler et al. (2017)
5	*O. andersonii*	SYS r001020	China, Guangdong, Shenzhen	KY594732	Wang et al. (2017)
6	*O. andersonii*	SYS r001382	China, Guangdong, Dongguan	KY594734	Wang et al. (2017)
7	*O. andersonii*	SYS r001423	China, Hong Kong	KY594730	Wang et al. (2017)
8	*O. cheni*	YBU071040	China, Hunan, Yizhang	GQ281779	Guo et al. (2012)
9	*O. cheni*	SYS r001422	China, Guangdong, Yingde	KY594741	Wang et al. (2017)
10	*O. daovantieni*	CIB 109024	Vietnam, Kon Tum, K Bang	PQ726905	Gao et al. (2024)
11	*O. durandi*	NCSM 80739	Vietnam	MK941137	Ziegler et al. (2019)
12	*O. guangxiensis*	GP746	China, Guangxi	GQ281776	Guo et al. (2012)
13	*O. haihaensis*	IEBR A.2016.34	Vietnam, Quang Ninh, Hai Ha	MK991139	Ziegler et al. (2019)
14	*O. haihaensis*	SYS r000537	China, Guangxi, Fangchenggang	MN890017	Wang et al. (2020)
15	*O. hungtai*	SYS r000538	China, Guangxi, Pubei	MN890018	Wang et al. (2020)
16	*O. hungtai*	SYS r000946	China, Guangdong, Fengkai	KY594748	Wang et al. (2017)
17	*O. jacobi*	IEBR 4329	Vietnam, Vinh Phuc, Tam Dao	MG545601	Ziegler et al. (2018)
18	*O. jacobi*	ZFMK 100818	Vietnam, Vinh Phuc, Tam Dao	MG545602	Ziegler et al. (2018)
19	*O. kuatunensis*	SYS r001008	China, Fujian, Shanghang	KY594746	Wang et al. (2020)
20	*O. kuatunensis*	SYS r001081	China, Guangdong, Shenzhen	KY594747	Wang et al. (2020)
21	*O. lateralis*	——	China, Guangxi	GQ281782	Guo et al. (2012)
22	*O. lateralis*	SYS r000951	China, Guangdong, Fengkai	KY594743	Wang et al. (2017)
23	*O. lateralis*	SYS r001080	China, Guangdong, Shenzhen	KY594744	Wang et al. (2017)
24	*O. lateralis*	ZFMK 100806	Vietnam, Bac Giang, Tay Yen Tu	MF477899	Ziegler et al. (2017)
25	*O. lateralis*	VNMN A.2016.14	Vietnam, Quang Ninh, Bai Tu	MF477900	Ziegler et al. (2017)
26	*O. latouchii*	SYS r000670	China, Fujian, Wuyishan	KY594742	Wang et al. (2017)
27	*O. latouchii*	GP647	China, Fujian	GQ281783	Guo et al. (2012)
28	*O. laui*	SYS r001161	China, Guangdong, Taishan	KY594739	Wang et al. (2017)
29	*O. laui*	SYS r001170	China, Guangdong, Taishan	KY594740	Wang et al. (2017)
30	*O. maculosa*	FMNH 265798	Thailand, Nong Khai, Phu Wua	MK991138	Ziegler et al. (2019)
31	*O. maxwelli*	SYS r000841	China, Guangdong, Nanao	KY594736	Wang et al. (2017)
32	*O. maxwelli*	SYS r001053	China, Fujian, Nanjing	KY594737	Wang et al. (2017)
33	*O. shenzhenensis*	SYS r001018	China, Guangdong, Shenzhen	KY594727	Wang et al. (2017)
34	*O. shenzhenensis*	SYS r001021	China, Guangdong, Shenzhen	KY594728	Wang et al. (2017)
35	*O. voquyi*	ZFMK 100819	Vietnam, Bac Giang, Tay Yen Tu	MG451049	Ziegler et al. (2018)
36	*O. voquyi*	ZFMK 100820	Vietnam, Bac Giang, Tay Yen Tu	MG451050	Ziegler et al. (2018)
37	*O. zhaoermii*	CIB109998	China, Hunan, Guzhang	MG012799	Ren et al. (2017)
38	*O. zhaoermii*	CIB109998	China, Hunan, Guzhang	MG012800	Ren et al. (2017)
39	*T. balteatus*	CIB 109018	China, Guangdong, Zhaoqing	MN017775	Ren et al. (2019)
40	*T. percarinatus*	CIB 109022	China, Fujian, Wuyishan	MN017779	Ren et al. (2019)

**Table 2.** Morphological data of the newly-collected *Opisthotropis tamdaoensis* specimens from China and from Vietnam, based on Ziegler et al. (2008, 2017), “—” means missing data.

	Chinese population		Vietnamese population		Range (total)
Voucher numbers	CIB 103778	CIB 103779			
Sex	Juvenile male	Female	adult males	adult females	
Numbers	n = 1	n = 1	n = 2	n = 3	n = 7
Tail length (TL)	231	558	537—555	456—522	456—558
Snout-vent length (SVL))	189	468	460—475	384—440	384—475
Tail length (TaL)	42	90	77—80	72—82	72—90
TaL/TL	0.182	0.161	0.143–0.144	0.157	0.143–0.161
Head length (HL)	8.73	13.26	—	—	—
Head width (HW)	4.05	11.28	—	—	—
Pre-oculars (PrO)	1	1	1-2	1-2	1-2
Postoculars (PtO)	2	2	2	2	2
Supralabials (SPL)	9/9	9/8	8–9	9	8–9
SPL–Orbit	5–6/5–6	5–6/5–6	5 (0, 5–6)	5–6 (5)	5 (0, 5–6)
Infralabials (IFL)	9/10	9/9	9–10	9–11	9–11
Temporals (TMP)	1+2/1+2	1+2/2+2	(1–2) + (3–4)	1 + (2–3)	(1–2) + (2–4)
Ventral scales (V)	165	160	171–176	162–165	160–176
Subcaudals (SC)	56	51	(49+)–51	48–50	48–56
Dorsal scale rows (DSR)	17-17-17	17-17-17	19-17-17	19-17-17	(17-19)-17-17

**Table 3.** Uncorrected *p*-distances (%) amongst *Opisthotropis* species, based on *Cytb*. Numbers refer to specimens listed in Table 1.

**Species**	**01-Apr**	**05-Jul**	**08-Sep**	**10**	**11**	**12**	**13-14**	**15-16**	**17-18**
1-4 *O. tamdaoensis*	0.5–2.9								
5-7 *O. andersonii*	13.1–15.2	1.2–4.5							
8-9 *O. cheni*	16.0–17.0	17.0–17.8	0.5						
10 *O. daovantieni*	19.2–19.5	16.1–16.8	17.7–17.8						
11 *O. durandi*	16.0–16.8	17.3–17.9	18.2–18.3	17.6					
12 *O. guangxiensis*	16.6–17.5	14.8–15.0	16.4–16.7	17.7	15.7				
13-14 *O. haihaensis*	14.9–16.4	15.6–16.5	15.9–16.0	18.1–18.6	17.1–17.3	14.6–14.7	4.4		
15-16 *O. hungtai*	16.3–17.4	16.3–16.8	17..3–17.6	18.7–19.0	17.4	15.7–16.1	13.0–13.5	2.8	
17-18 *O. jacobi*	17.2–17.7	15.9–16.4	14.8–15.0	16.4–16.5	15..6–15.8	13.6–13.8	13.9–14.3	14.0–14.6	0.4
19-20 *O. kuatunensis*	15.3–17.0	16.1–16.8	14.1–14.5	17.4–18.0	17.3	15.9–16.0	16.3–16.8	15.7–16.6	15.0–15.3
21-25 *O. lateralis*	5.9–6.7	14.1–14.8	15.6–16.4	18.7–19.1	16.9–17.2	16.8–17.1	16.0–16.5	17.4–18.7	17.1–17.6
26-27 *O. latouchii*	15.5–16.1	16.0–16.4	5.0–5.1	17	17.4	15.6	15.4–16.1	16.6–16.8	14.8
28-29 *O. laui*	13.4–14.6	12.2–12.8	16.6–16.8	17.9–18.0	17.8–17.9	16.4–16.5	15.3–16.2	15.3–16.7	15.4–15.6
30 *O. maculosa*	17.1–18.0	18.5–18.7	17.7	18.7	16.8	16	16.2–17.3	17.9	15.7–15.8
31-32 *O. maxwelli*	13.1–14.8	11.2–12.5	15.9–16.6	17.5–18.2	17.1–17.8	15.6–15.9	15..1–16.1	14.8–15.4	15.1–16.1
33-34 *O. shenzhenensis*	13.7–15.4	11.0–11.8	17.1–17.4	18.4–18.5	18.0–18.2	16.5–16.7	17.3–17.8	16.4–16.6	16.1–16.4
35-36 *O. voquyi*	14.5–15.3	14.4–14.6	14.4–14.8	16.7–16.8	15.3–15.4	13.3–13.4	13.3	14.8–15.0	9.9–10.2
37-38 *O. zhaoermii*	15.7–16.5	16.0–16.2	5.6	18	17.9	16	15.6–15.8	16.8–17.1	14.2
**Species**	**19-20**	**21-25**	**26-27**	**28-29**	**30**	**31-32**	**33-34**	**35-36**	**37-38**
19-20 *O. kuatunensis*	1.1								
21-25 *O. lateralis*	16.5–17.1	0.4–2.5							
26-27 *O. latouchii*	13.8–14.1	15.9–16.1	0						
28-29 *O. laui*	16.5–16.8	14.9–15.6	16.3–16.4	0.1					
30 *O. macculosa*	16.5–16.6	17.6–18.0	17.1	18.6–18.7					
31-32 *O. maxwelli*	15.4–16.2	13.3–14.5	15.6–16.0	11.4–12.0	17.4–17.6	1.5			
33-34 *O. sheenzhenensis*	17.4–18.0	14.6–15.2	16.4–16.6	14.1–14.4	18.2–18.4	11.0–11.6	0.3		
35-36 *O. voquyi*	15.6–15.7	14.6–15.0	14.1–14.2	13.6–13.7	16.2–16.3	14..4–15.3	14.9–15.1	0.1	
37-38 *O. zhaoermii*	14.8	16.1–16.4	5.1	16.5–16.6	17.5	15.3–15.9	16.1–16.3	14.5–14.6	0
